# Perceiving ensemble statistics of novel image sets

**DOI:** 10.3758/s13414-020-02174-0

**Published:** 2021-01-08

**Authors:** Noam Khayat, Stefano Fusi, Shaul Hochstein

**Affiliations:** 1 ELSC Edmond & Lily Safra Center for Brain Research and Silberman Life Sciences Institute, Hebrew University, Jerusalem, Israel; 2 Mortimer B. Zuckerman Mind Brain and Behavior Institute and Department of Neuroscience, Columbia University, New York, NY USA

**Keywords:** Categorization, Implicit/explicit memory, Visual perception, Ensemble Perception

## Abstract

Perception, representation, and memory of ensemble statistics has attracted growing interest. Studies found that, at different abstraction levels, the brain represents similar items as unified percepts. We found that global ensemble perception is automatic and unconscious, affecting later perceptual judgments regarding individual member items. Implicit effects of set mean and range for low-level feature ensembles (size, orientation, brightness) were replicated for high-level category objects. This similarity suggests that analogous mechanisms underlie these extreme levels of abstraction. Here, we bridge the span between visual features and semantic object categories using the identical implicit perception experimental paradigm for intermediate novel visual-shape categories, constructing ensemble exemplars by introducing systematic variations of a central category base or ancestor. In five experiments, with different item variability, we test automatic representation of ensemble category characteristics and its effect on a subsequent memory task. Results show that observer representation of ensembles includes the group’s central shape, category ancestor (progenitor), or group mean. Observers also easily reject memory of shapes belonging to different categories, i.e. originating from different ancestors. We conclude that complex categories, like simple visual form ensembles, are represented in terms of statistics including a central object, as well as category boundaries. We refer to the model proposed by Benna and Fusi (*bioRxiv* 624239, 2019) that memory representation is compressed when related elements are represented by identifying their ancestor and each one’s difference from it. We suggest that ensemble mean perception, like category prototype extraction, might reflect employment at different representation levels of an essential, general representation mechanism.

## Introduction

It has been widely reported that we perceive the mean and range of image sets. This has been found for basic image parameters such as circle size, disc brightness, line orientation, image color, motion speed, and direction, and for more complex variables such as face emotion, identity, and lifelikeness (see *Background*, below). We recently extended the study of the ensemble statistics phenomenon to include the realm of unconscious perception, finding that observers base task judgements on the automatically, unconsciously perceived mean of a recent past sequence of images (Khayat & Hochstein, 2018). Furthermore, we found similarity between characteristics of set range and mean perception and image category and prototype perception (Khayat & Hochstein, 2019a). But categorization is generally of well-known categories learned over lifetime experience. It is also generally a more cognitive, semantic phenomenon, while the basic features tested in ensemble statistics perception were more sensory and not semantic. We now bridge the gap between categorization and set statistics perception by testing perception of the mean and category of novel image categories, learned “on-the-fly” while viewing a single sequence of category exemplars.

A second goal of the current study was to enable direct measurement of category member proximity to or distance from the category mean or prototype. In our previous study relating categorization to set perception, we used an auxiliary experiment to test category exemplar typicality (Khayat & Hochstein 2019a). In that experiment, we presented two test images, one a member of a previously named category and one of a different category, and measured the speed by which observers judged which image was the member of the named category. The faster the response, the closer to the prototype was the object image considered. While this is an accepted methodology for determining object category typicality (e.g., Ashby & Maddox, 1994; Rosch, Simpson, & Miller, 1976), and its appropriateness was confirmed by the consistency of the experimental results, it is indirect, may be culturally and semantically determined, and may not reflect the same aspect of typicality as measured in the sequence memory test. The current study was thus designed to allow direct measures of distance from the category mean.

**Fig. 1:**
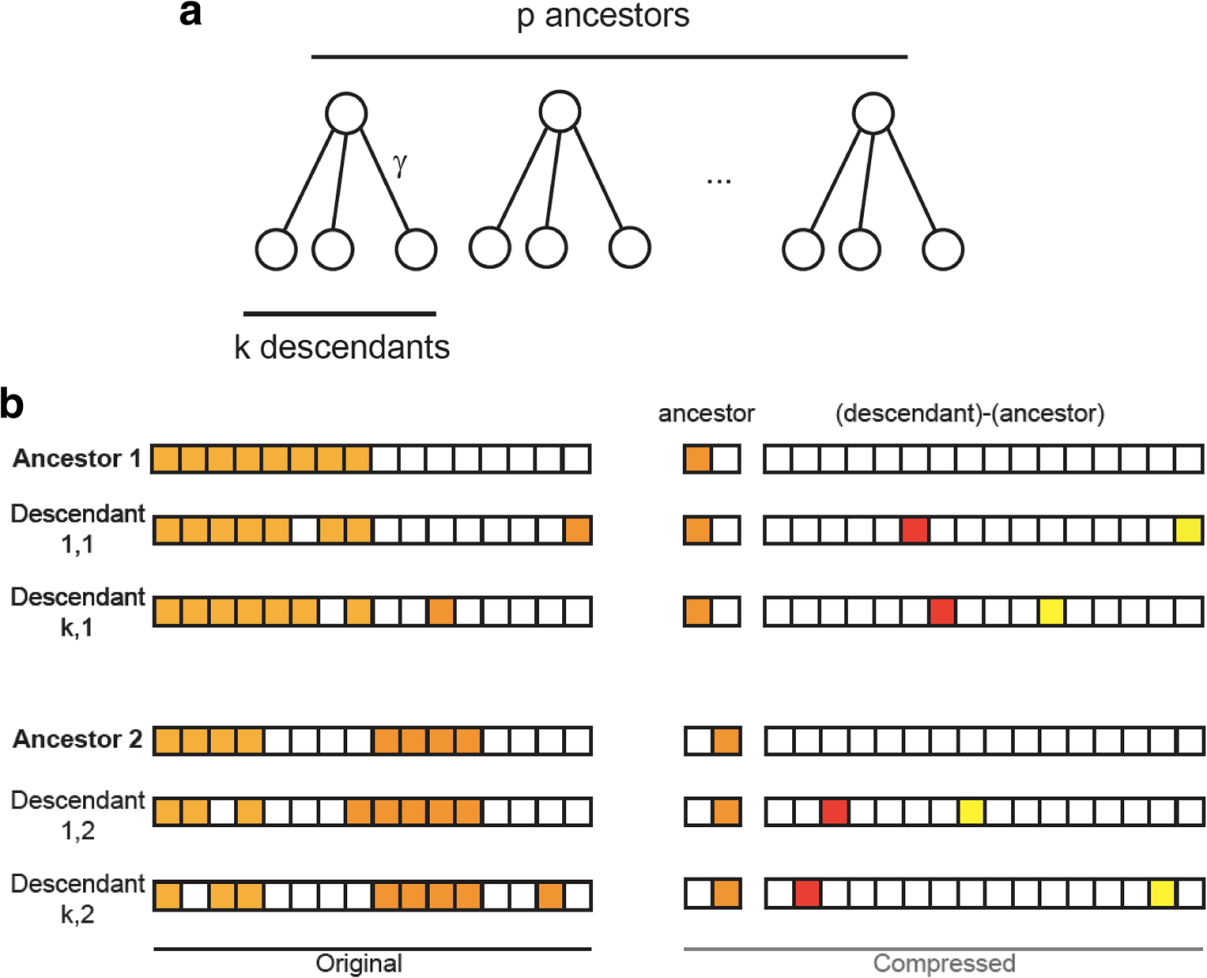
Storing correlated patterns. (**a**) Schematic representation of an ultra-metric tree with p ancestors and k descendants per ancestor used to generate correlated patterns. (**b**) Possible scheme using correlations to generate compressed representations that are sparse and more efficiently storable. From Benna and Fusi (2019)

The third goal relates to a recent paper by Benna and Fusi (2019) concerning efficient representation of correlated patterns (see also Schapiro & Turk-Browne, 2015). Most patterns stored in memory are highly correlated with others, as we often have similar experiences. Storing correlated patterns in their original format is not optimal, and capacity can be increased by taking the correlations into account. For example, patterns may be organized in an (ultrametric) tree, as in Fig. 1a (see, e.g., Rammal et al., 1986). If we start with a small set of uncorrelated patterns (the ancestors at the top of the tree) and generate numerous descendants for each ancestor (by modifying activation of some ancestor-representing neurons), then the descendants of each ancestor are correlated by their similarity to the ancestor. Benna and Fusi suggest that efficient representation of such patterns is by storing the identity of the appropriate ancestor, and the difference between it and each descendant, as in Fig. 1a. By construction, the ancestor-descendant differences are small and approximately uncorrelated. A more general compression strategy that can be applied to any set of correlated patterns (not only those organized in an ultrametric tree) is based on sparse auto-encoders (Olshausen & Field, 1996). We wished here to determine if using stimulus patterns that are related in such an ancestor-descendant fashion would lead to automatic perception of the ancestor pattern, irrespective of whether it is presented in an image sequence.

## Background

**Fig. 2:**
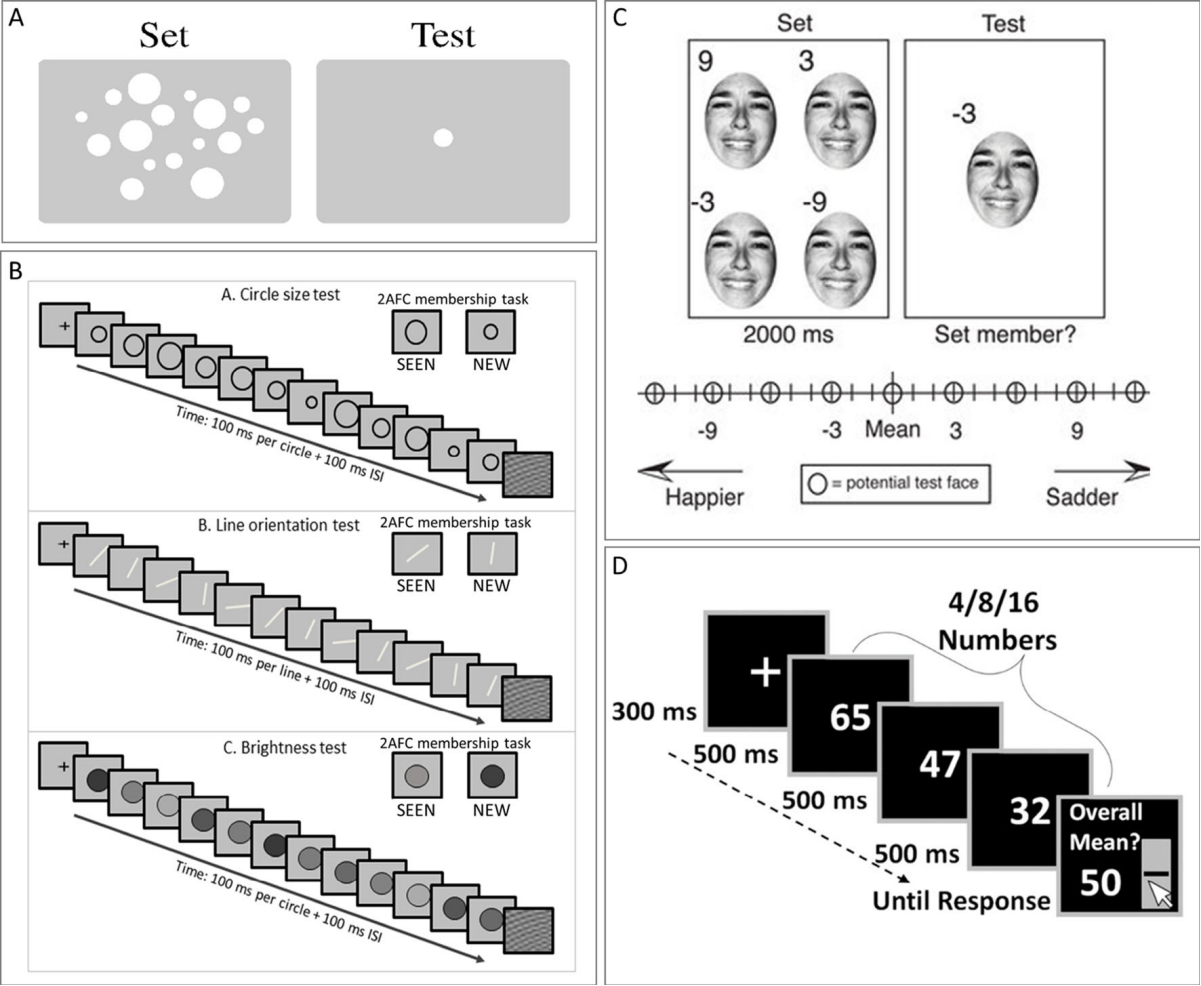
Previous study stimulus sets. (**A**) Ariely’s (2001) schematic representation of the two intervals used in his experiment’s trials. Observers were exposed for 500 ms to a set of spatially dispersed circles differing by size and then asked if a test stimulus size had been present in the set, or is smaller/larger than the set mean. (**B**) Khayat and Hochstein’s (2018) RSVP sequences consisted of 12 elements, each presented for 100 ms plus a 100-ms inter-stimulus interval (ISI), followed by a two-alternative forced-choice (2-AFC) membership test (i.e., which test element had been present in the sequence). Blocks contained circles differing in size, lines differing in orientation, or discs differing in brightness. Observers were asked which of two test elements was present in the set. They were unaware that either test element could equal the set mean or the nonmember could be outside the set range. (**C**) Haberman and Whitney’s (2009) task included four faces (from a set of 4, 8, 12, or 16), differing in facial emotional expression, presented for 2 s. Observers then indicated whether the test face was a member of the set, or was happier/sadder than the set mean. (**D**) Brezis et al.’s (2015) trials consisted of two-digit numbers sequentially presented at a rate of 500 ms/stimulus. Set size was 4, 8, or 16. Participants estimated set average

There has been a great deal of recent interest in perception of summary statistics of sets of stimulus elements. Observers reliably represent the mean and range of stimulus sets, even without reportable perception of individual set members. They rapidly extract summary statistics from sets of similar items, when these are presented spatially (Alvarez & Oliva, 2009; Ariely, 2001) or temporally (Corbett & Oriet, 2011; Gorea, Belkoura, & Solomon, 2014; Hubert-Wallander & Boynton, 2015). Statistics include average, and range or variance of their size (Allik, Toom, Raidvee, Averin, & Kreegipuu, 2014; Ariely, 2001; Corbett & Oriet, 2011; Khayat & Hochstein, 2018; Morgan, Chubb, & Solomon, 2008; Solomon, 2010), orientation (Alvarez & Oliva, 2009; Khayat & Hochstein, 2018; Hochstein, Pavlovskaya, Bonneh, & Soroker, 2018), brightness (Bauer, 2009; Khayat & Hochstein, 2018), spatial position (Alvarez & Oliva, 2008), and speed and direction of motion (Sweeny, Haroz, & Whitney, 2013). Thus, summary statistics extraction appears to be a general mechanism operating on various stimulus attributes, including low-level information, as mentioned above, and more complex characteristics, such as facial expression (emotion) and gender (Haberman & Whitney, 2007, 2009; Neumann, Schweinberger, & Burton, 2013), object lifelikeness (Yamanashi-Leib, Kosovicheva, & Whitney, 2016), biological motion of human crowds (Sweeny, Haroz, & Whitney, 2013), and numerical averaging (Brezis, Bronfman, & Usher, 2015; for recent reviews, see Bauer, 2015; Cohen et al., 2016; Haberman & Whitney, 2012; Hochstein, Pavlovskaya, Bonneh, & Soroker, 2015). Examples of methods used in these studies are shown in Fig. 2.

We suggested that the cerebral mechanism for categorization may be related to that underlying set perception since they share basic characteristics (Hochstein, 2016a, 2016b; Khayat & Hochstein, 2019a). In both cases, when viewing sets of similar items, we consider them the same, as a shortcut to representation and prescription of an appropriate response (Ariely, 2001; Medin, 1989; Rosch & Mervis, 1975; Rosch, Mervis, et al., 1976). Globally spreading attention, we see a line of cars, shelf of bottles, flock of sheep, or copse of trees. We then categorize these objects and relate to the average properties of each category. Similarly, laboratory experiments, present a set of circles (Alvarez & Oliva, 2008; Ariely, 2001; Corbett & Oriet, 2011; Khayat & Hochstein, 2018), line segments (Khayat & Hochstein, 2018; Robitaille & Harris, 2011), or faces (Haberman & Whitney, 2007, 2009), and observers perceive the images as circles, lines, or faces and relate to their average properties. Categorization emphasizes relevant or common properties, de-emphasizing irrelevant or uncommon properties (Fabre-Thorpe, 2011; Goldstone & Hendrickson, 2010; Hammer, Diesendruck, Weinshall, & Hochstein, 2009; Rosch, Mervis, et al., 1976; Rosch, 1973, 1999, 2002; Rosch & Lloyd, 1978). Similarly, set perception captures summary statistics without noting individual values. Categorization, like ensemble perception, may depend on rapid feature extraction to determine presence of defining object characteristics. Set perception includes mean and range (Ariely, 2001; Chong & Treisman, 2003, 2005; Khayat & Hochstein, 2018; Hochstein et al., 2018), and categorization might rely on the related properties of prototype (or mean exemplar; e.g., Ashby & Maddox, 2011) and inter-category boundaries (or category range; e.g., Goldstone & Kersten, 2003). We confirmed this conceptual similarity, finding that set characteristics are perceived implicitly and automatically (Khayat & Hochstein, 2018), just as objects are categorized implicitly and automatically at their basic category level (Potter & Hagmann, 2015; Rosch, Mervis, et al., 1976). It has also been suggested that determining whether objects belong to a single category may depend on the same characteristics that define them as a set (Utochkin, 2015). Finally, our studies showed that the detailed properties of set and category perception are similar (Khayat & Hochstein, 2019a), suggesting that analogous mechanisms might be responsible for their cerebral representation. This analogy is detailed below.

### Previous studies

We studied implicit perception and memory of set statistics by presenting a rapid serial visual presentation (RSVP) sequence of images differing by low-level properties (circles of different size, lines of different orientation, discs of different brightness; see Fig. 2B), and testing only memory of the members seen in the sequence (Khayat & Hochstein, 2018). The mean of the set – mean size circle, mean orientation line, or mean brightness disk – was sometimes included in the set sequence. Following set RSVP presentation, we presented two images simultaneously, side by side, one SEEN in the sequence and one a NEW item. We tested observer memory by asking participants to choose which test image had been SEEN in the sequence. We did not inform them that one test element could be the sequence mean, whether the SEEN test item (i.e., a RSVP sequence member) or the NEW foil item, (i.e., not a sequence member). Also, we did not inform them that sometimes the NEW test image was outside the sequence range. We purposely did not mention the words “mean” and “range,” so that we could test if observers automatically perceive set mean and choose test items that match the mean. We also asked if observers would automatically perceive set property range and easily reject foils outside the sequence range.

We call these test-stimulus contingencies trial subtypes, as shown in Table 1, using the following terms: “in” and “out” – test elements within and outside the range of the variable sequence property; “mean” – element with property equal to mean of sequence.

**Table 1:** Test image (subtype) contingences and expected results

SEEN test element Correct	NEW test element Incorrect	Expected performance
In range	Out of range	Best
Mean	In range	Better
In range	In range	Baseline
In range	Mean	Worse

**Fig. 3:**
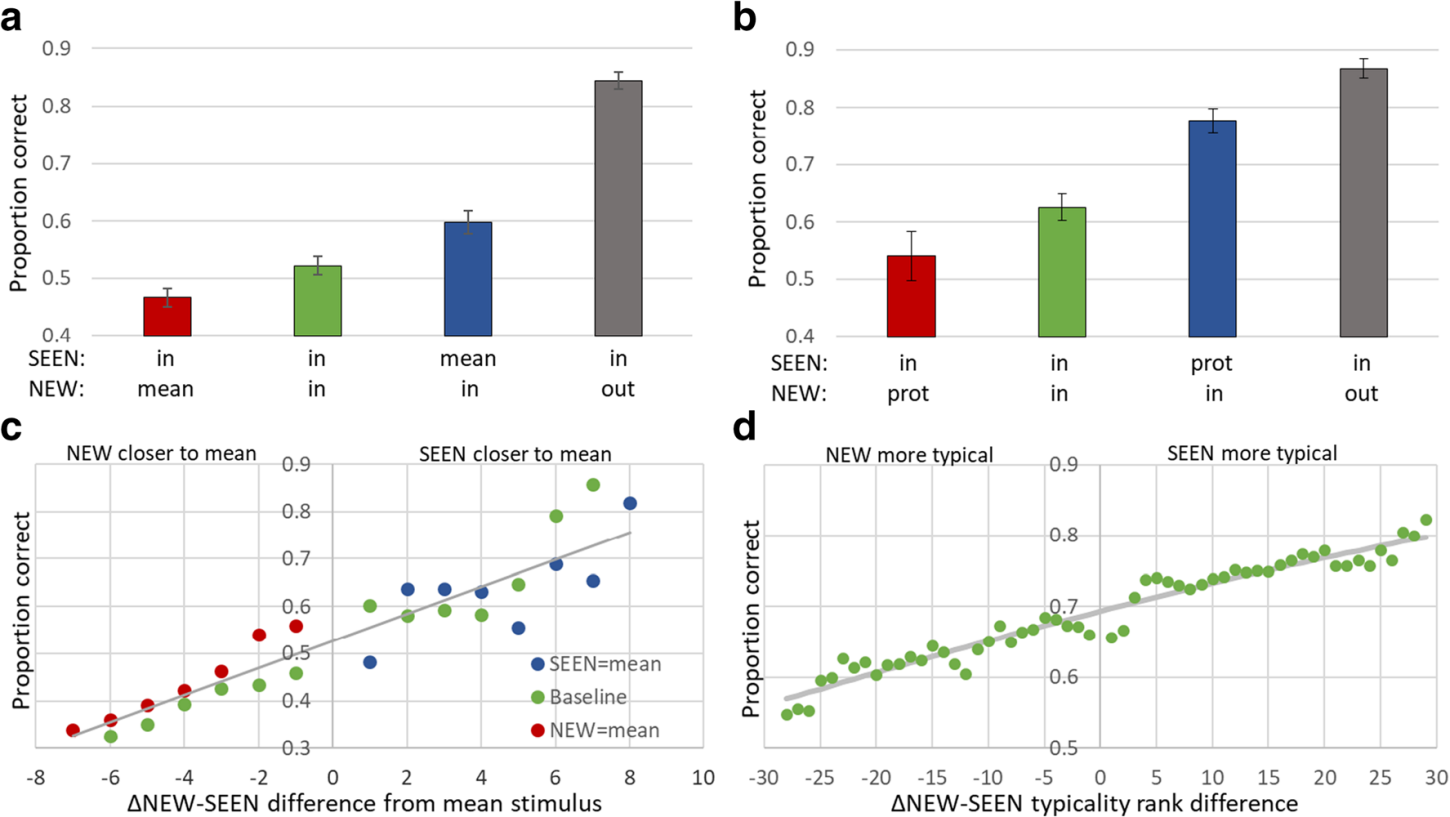
Low-level parameter mean perception (**a, c**) compared to category prototype perception (**b, d**). Participants viewed a sequence of images varying in a low-level parameter (Fig. 1b), i.e., circle size, line orientation, or disc brightness (**a, c**), or a sequence of object images from a single category (**b, d**), followed by two test images, one SEEN in the sequence and one NEW. They were asked to choose the SEEN image. Participants had difficulty remembering sequence images. Instead, the SEEN (graph blue bar) or NEW (red) image that matched the mean or category prototype was preferred, relative to the case where neither test image matched the mean or prototype (green) and a NEW image from outside the range or from a different category was rejected (black). See text. From Khayat and Hochstein (2018, 2019a). This preference was graded in that the closer the test image to the mean (**c**) or the greater its category typicality (**d**), the greater the chance of its being chosen as SEEN, as measured by probability of choice dependence on the difference between the test images’ distance from the mean (**c**) or difference in their typicality (**d**). prot = prototypical object image, in = in range, out = out of range, mean = ensemble mean. Error bars in all figures indicate standard error of the mean. Differences in mean accuracy between all pairs of trial subtypes in both low-level and categorization studies were significant, p < 0.05

We expected, as then found, that it would be difficult for participants to perceive, represent, and remember all the images in each sequence, and that they would depend, instead, on implicit perception of the sequence mean to direct their choice of test image. Thus, as displayed in Table 1, when neither image is the mean, baseline performance would be low, and when one of the test images is the mean, it will be preferred. On the other hand, an image that is outside the range of sequence images would be more easily rejected. This is what occurred, as demonstrated in Fig. 3a. Furthermore, perception of the sequence mean was graded in that elements closer to the mean were preferred and this preference grew as the difference between the distances of the SEEN and NEW element test images from the mean grew, as shown in Fig. 3c.

The same pattern of results was found for sequences of images of objects belonging to a single category. Some perception of the objects was maintained in memory, but when one of the test object images was the category prototype, it was preferred, as shown in Fig. 3b, whether it was the SEEN or the NEW object. Implicit perception of each trial’s sequence category and its prototype led to a graded preference for the object image that was more typical of the category, as demonstrated in Fig. 3d. The categorization experiment results all show greater accuracy than the low-level feature experiment, presumably because participants remember some individual pictures of real objects. Nevertheless, the similarity of Fig. 3a and b and of Fig. 3c and d suggests that analogous mechanisms might underlie both phenomena – ensemble mean perception, on the one hand, and object categorization and category prototype derivation, on the other.

Noting that the categories of the categorization experiment were learned over a lifetime of experience, while the low-level parameter sequences were learned on-the-fly, we sought an intermediary test of categories that could be learned on-the-fly, trial by trial. In addition, the graded measure of typicality in the categorization test depended on an auxiliary experiment to determine each object’s typicality (see Khayat & Hochstein, 2019a), and we wish to test categorization where typicality is directly measurable. These were central goals of the current set of experiments, as outlined in the *Introduction* and described in the *Methods*.

## Methods

### Design and stimuli

**Fig. 4:**
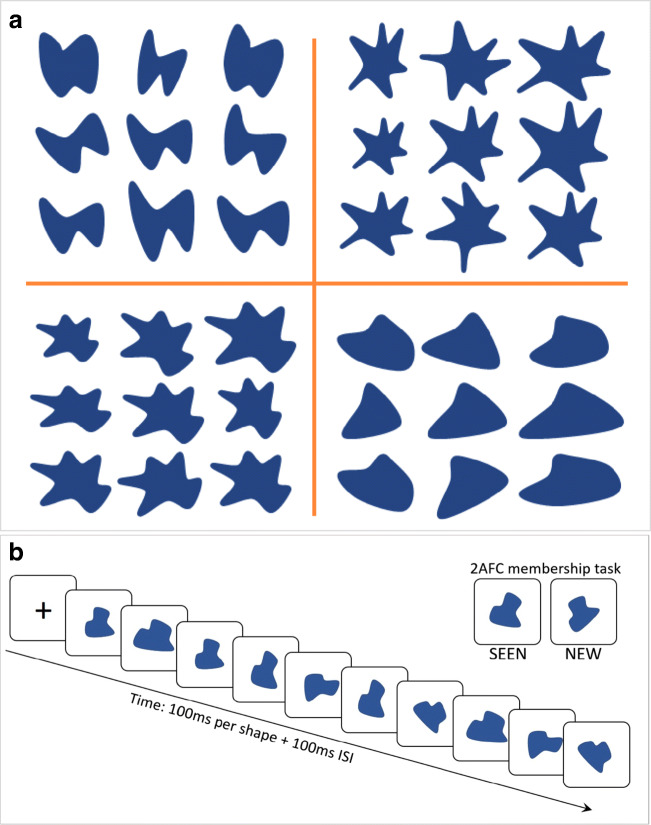
(**a**) Examples of random amoeba shapes for Experiment 1. The central image is the ancestor and the surrounding eight images are descendants, created by applying distortions to the ancestor, enlarging or compressing, rotating, stretching, or shrinking. (**b**) Trial design. RSVP sequences of ten elements, followed by a two-alternative forced-choice (2-AFC) membership test, asking which test element had been present in the sequence

We performed five sets of experiments with novel “amoeba” shapes (e.g., Fig. 4a). Precise construction of the shapes, the ancestors, and their descendants are described below for each experiment. The general design of all the experiments was the same as for the low-level and categorization experiments described above (Khayat & Hochstein, 2018, 2019a; Figs. 2B and 3, Table 1). As demonstrated in Figs. 4b, 6d, and 8, on each trial a sequence of eight stimuli was presented in RSVP in the center of the monitor screen with an exposure time of 100 ms/stimulus and a 100-ms ISI (in Experiment 1, sequences included five images, each presented twice), all in random order. Participants were instructed to remember all the stimuli to perform the two-alternative-forced choice (2-AFC) task, which followed. Here, two stimuli were presented side by side, one which was SEEN in the sequence and one which was NEW; presentation was until response. Participants were instructed to indicate which stimulus was a member of the sequence by pressing the left (NEW image) or right (SEEN image) arrow on the keyboard. The stimuli of each trial’s sequence belonged to a single ensemble, as described below.

The role of the new aspect in the current experiments is twofold: first, we mimic the ancestor-descendent tree suggested by Benna and Fusi (2019); in addition, we create new categories that participants can learn on-the-fly, on a trial-by-trial basis. In each RSVP ensemble sequence, all member exemplars are descendants of one ancestor, including, on some trials, the ancestor itself. Examples of sets of Experiment 1 amoeba shapes are shown in Fig. 4a, where in each case the central image is the ancestor and the surrounding eight images are descendants.

We did not tell the participants that the sequence sometimes included the ancestor. Furthermore, one of the two side-by-side test images, SEEN or NEW, could be the ensemble ancestor. If the ancestor was included in the sequence, then it could be the SEEN test image; if it was not included in the sequence, it could be the NEW test image. Of course, SEEN and NEW test images could also both be descendants. Finally, sometimes a descendant of another ancestor was used as the NEW test image. Participants were not informed of these test image conditions and were assumed to be naïve to involvement of ancestor, descendant, or any other ensemble statistics. Any effect of these measures on performance would thus be implicit.

The task requested of the participants was to remember the images presented in the RSVP sequence. However, we expect participants to have difficulty remembering them, and instead to depend on their implicit perception of the ancestor – whether presented or not. Thus, if neither test image is the ancestor, performance accuracy should be close to 50%. If the SEEN image is the ancestor, accuracy (fraction of correctly identifying the SEEN images) should be larger, and if the NEW image is the ancestor, it may falsely be chosen as if seen, and accuracy should drop. Finally, if one test image is from a different ancestor, it should be more easily rejected and accuracy should be enhanced. The alternative test subtypes are shown in Table 2. For Experiments 1–3, 20 different trials of each subtype were presented to each participant; for Experiment 4, 24 trials each, and for Experiment 5, 64 trials each.

**Table 2:** Test image (subtype) contingences and expected results for all experiments

SEEN test element Correct	NEW test element Incorrect	Expected performance
Descendant	Descendant of different ancestor	Best
Ancestor	Descendant	Better
Descendant	Descendant	Baseline
Descendant	Ancestor	Worse

### Participants

Experiments were conducted using the Amazon Mechanical Turks (MTurks) website, a crowdsourcing platform enabling coordination of online participants of uploaded human cognitive tasks, using Adobe Flash. Participants provided informed consent and received compensation. Participants were naïve as to the purpose of the experiment, and, as mentioned above, were not aware of test image contingencies such as ancestor, descendant, or category.

We had little control of experimental conditions, but performance itself indicates whether participants were trying to perform the task or were pressing responses randomly. We excluded data of participants whose overall accuracy was below 55%, and any trial where response time (RT) was below 200 ms or longer than 3 s. We tested 600 MTurks for the five experiments reported here, excluding data of 24 (Experiment 1: 50 participants; Experiment 2: 150, of which five were excluded; Experiment 3: 100, ten excluded; Experiment 4: 100, nine excluded; Experiment 5: 200, no exclusions).

### Data analysis

One-way repeated-measure analysis of variance (RM-ANOVA) was conducted to verify that performance accuracy differences were due to biases to the ensemble characteristics of membership test stimuli (e.g., similarity to ensemble ancestor) in different trial subtypes, rather than participant differences in performance. Variance differences of ancestor effects were measured (within subjects) by three trial subtypes (independent variables; baseline trial vs. trials where the SEEN or NEW test stimuli were equal to the ancestor). Variance between baseline trials and trials where the NEW was derived from a different ancestor was measured to evaluate effect of ensemble range. Additionally, (one-tailed) t-tests between the averaged results over participants for different subtype combinations were performed to investigate ancestor and range representation effects. Some of these experiments have been reported previously in meeting abstract or brief communication format (e.g., Hochstein, 2019; Hochstein et al., 2018, 2020; Hochstein, Khayat, Pavlovskaya, Bonneh, & Soroker, 2019; Hochstein, Khayat, Pavlovskaya, Bonneh, Soroker, & Fusi, 2019; Khayat & Hochstein, 2019b).

In the Appendix we present violin plots of the data for each experiment.

## Results

### Experiment 1: Sets of random amoeba shapes

To mimic the ancestor-descendent tree suggested by Benna and Fusi (2019), we drew random shapes in PowerPoint – the ancestors – and then applied various distortions to the shapes to create descendants. Distortions included enlarging or compressing, rotating, and stretching or shrinking in one dimension. Examples of sets of random amoeba shapes are shown in Fig. 4a, where in each case the central image is the ancestor and the surrounding eight images are descendants. Each trial had a sequence of ten images (five different images presented twice each, in random order), derived from a new ancestor, and thus new descendants, so that each image was only part of a single sequence.

**Fig. 5:**
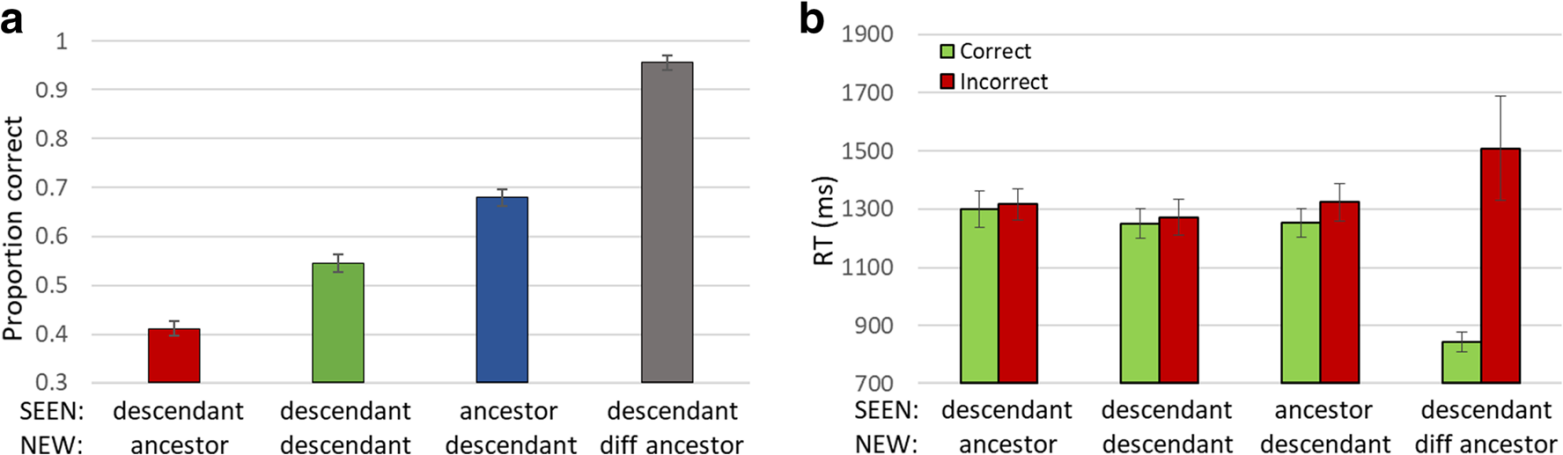
Experiment 1 – random amoeba images. (**a**) Perception and memory of the ancestor, whether present (blue) or absent (red) steers performance above or below baseline (green), respectively. Rejecting images from another ancestor improves performance dramatically (grey). Thus, participants clearly perceive the amoeba forms as a set with a definite ancestor image from which they descend. Differences in mean accuracy between each two trial subtypes were significant (p < 0.001). (**b**) Reaction time (RT) for different trial subtypes, for correct (green) and incorrect (red) responses, for each trial type

Performance for the amoeba ancestor-descendant experiment adhered to the expectations of Table 2, as shown in Fig. 5. Performance for baseline, where both SEEN and NEW test elements were descendants, was 0.54 ± 0.017 (average ± S.E.M., here and throughout; Fig. 5a, green bar), slightly but significantly above 50% (t-test; p < 0.01), suggesting that there was some, though little, memory of the SEEN images. There was considerable dependence on perception and memory of the ancestor, as shown by RM-ANOVA on the three relevant trial subtypes (here and in subsequent experiments): baseline and trials where SEEN or NEW shapes were the ensemble base, (F(2,98) = 70.59, p < 0.001), so that when the SEEN element was the ancestor, performance was elevated to 0.78 ± 0.016 (Fig. 5a, blue bar; t-test: p < 0.001; all t-tests are across participants, relative to baseline where SEEN and NEW are both descendants). When the NEW element was the ancestor, preferring it lowered performance (measured always as choice of the SEEN element), to 0.41 ± 0.014 (Fig. 5a, red bar; t-test: p < 0.001). We use as a general measure of ancestor perception the difference between performance when the SEEN image is the ancestor (raising performance) and when the NEW image is the ancestor (reducing performance). Here that is 0.67–0.41 = 0.26 (t-test difference: p < 0.001).

Performance when the NEW element was a descendant of a different ancestor (range effect, when the SEEN element was a descendant, Fig. 5a, grey bar) was elevated to 0.95 ± 0.014 (RM-ANOVA comparing NEW element originating from same ancestor, i.e. baseline, or different ancestor; here and in subsequent experiments: F(1, 49) = 327.09, p < 0.001). Thus, participants easily rejected elements from a different ancestor. This was confirmed by the RT measure (Fig. 5b) showing no speed-accuracy trade-off. While generally longer responses are expected for incorrect choices (not significant here), we found this gap was greatly increased for the easier condition, when the NEW test shape was from a different ancestor (t-test p < 0.005).

We conclude that for random amoeba-like ancestor shapes, distorted to create sets of descendants, participants perceive these images as belonging together, that is, as forming a set or category. Furthermore, they easily recognize the ancestor shape and remember it better than the descendants. In addition, they form a representation of the ancestor even when it has not been seen together with a set of descendants, and recall this never-seen image better than each of the seen descendants.

### Experiment 2. Sets of star-like amoeba shapes

#### Methods – Experiments 2–5

The goal of Experiments 2–5 was to produce amoeba shapes more systematically, allowing for measured distances between ancestor and descendant. In an ideal ultrametric tree, there are equal distances of each descendent from the ancestor. For example, if the ancestor is represented by a pattern of activity in **n** neurons, then each ancestor might have the activity of exactly **m** neurons changed from those of the ancestor, with most neurons, **n-m**, remaining unchanged. Here we start with star-like amoeba shapes, which are very different, and make relatively small changes to form descendants.

**Fig. 6:**
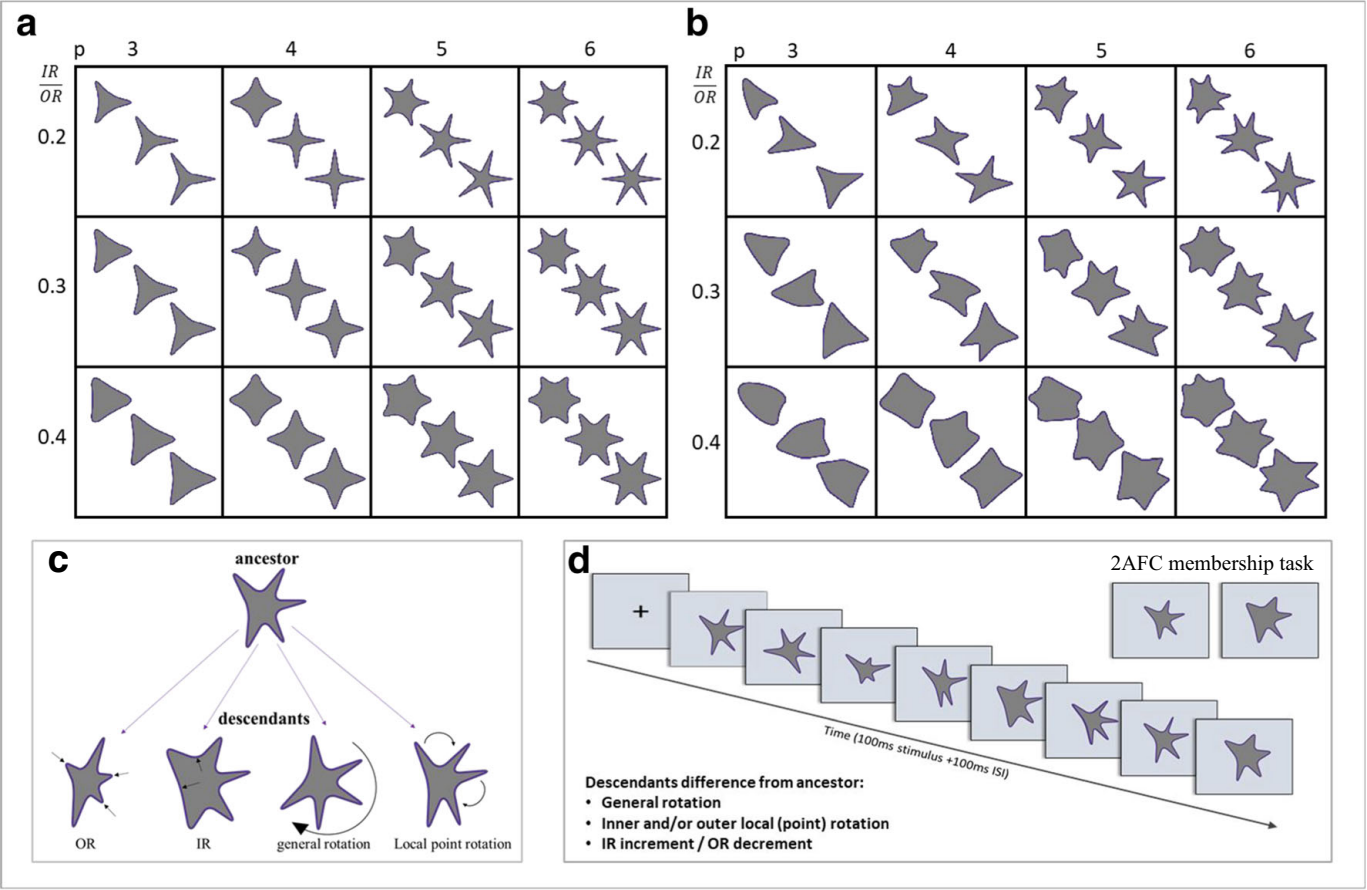
Constructing ancestor and descendants of star-like Amoeba shapes. (**a**) Equidistant symmetric star-like shapes defined by number of corners, p, across; inner:outer vertex radius ratio, IR:OR, down; and sharpness-roundness level, within each square. (**b**) Examples of ancestors, constructed from symmetric star-like shapes by general rotation; rotating local outer vertices individually; and shifting local vertex radii inward or outward (decreasing and increasing them, respectively). (**c**) Illustration of ancestor modification for Experiment 2, creating its descendants by increasing or decreasing local Inner or outer vertex radii, respectively, general rotation and/or local vertex point rotation. (**d**) Illustration of Experiment 2 trial paradigm: RSVP (100 ms/shape + 100-ms ISI; up to two changes/stimulus) followed by a two-alternative forced-choice (2-AFC) membership task

Experimental procedure was the same as for Experiment 1, except that the stimuli were all star-like images. We chose star-like amoeba shapes with p = 3–6 outer points or vertices. Stimuli for Experiments 2–5 were generated using Psychtoolbox Version 3 for MATLAB. Construction of the stars was as follows: We chose two circles with radii OR (outer radius = half of the width or height of the full used portion of the screen) and IR (inner-radius = 0.2, 0.3, or 0.4 times OR) and drew p equidistant points on the outer circle beginning with 0^o^ at the rightmost point on the circle, and p points half-way (in angular degrees) between each pair, on the inner circle. We then drew q (= 1, 3, or 6) equidistant dots between each outer point and the closest inner points; connecting these constructs the star. To have the star less pointy, i.e. with more rounded outer and inner vertices, we used the Python Pycairo library 1.18.1 smooth-connect function to connect the dots. Figure 6a demonstrates this procedure and shows examples of such equidistant-point stars.

Star amoeba ancestors were constructed beginning with one of the equidistant stars (choosing the number p of points, and the inner:outer radius ratio, IR:OR), rotating the entire star by an angle θ, where -90/p < θ < +90/p. Then, to render the star points non-equidistant, we shifted each outer vertex, i, by angle γ_i_, where -180/p < γ_i_ < 180/p. Inner radius points were shifted to remain halfway between the closest outer points. Then each outer vertex was shifted inward, towards the inner circle, by 0.25–0.5 times (OR–IR) and each inner vertex was shifted outward towards the outer circle, increasing its radius to 1.5–1.75 times IR. Examples of ancestor stars are shown in Fig. 6b.

For each ancestor star, we constructed up to a dozen descendants by changing one or two of the following parameters: general rotation of the star (by ±20^o^ or ±40^o^); rotation of some points along their circles (one point by ±10^o^ or half the points (p/2 points for even p; (p+1)/2 for odd p) by ±15^o^); changing the radial distance of some points (one point or half the points) from the OR (×0.6 or 0.55) or IR (×2.0 or 2.2) circles. Note that these changes were small compared to the differences between ancestors (changes were slightly different for the first 50 and last 95 participants, as indicated; differences between the results were insignificant). Examples of ancestors and their descendants for Experiment 2 are shown in Fig. 6c and in the trial illustration in Fig. 6d.

It became clear, both from computation and from experience with pilot experimentation, that the ancestor image drawn in this fashion, and used to construct the descendants, was not equal to the mean of the descendants. For example, if some descendants were rotated clockwise and none were rotated counter-clockwise, then the average was necessarily more clockwise than the base. It is possible, even likely, that participants derive as the set ancestor, the mean of the images seen in the sequence, rather than the base we used to create the descendants. We therefore tested for perception and memory of both base and mean images, compared with memory of descendant images presented in the RSVP sequence. To this end, for each sequence, we computed the average position of each star vertex, and use this as the mean shape. Thus, in Table 2, wherever the term “ancestor” appears, we conducted two tests, once with the base, and once with the computed mean. Tests for NEW = different ancestor included SEEN = descendant, mean or base; since these are relatively close, we present data here only for SEEN = descendant. In Experiment 5, below, we construct descendant sets where their mean was exactly the ancestor.

#### Results

**Fig. 7:**
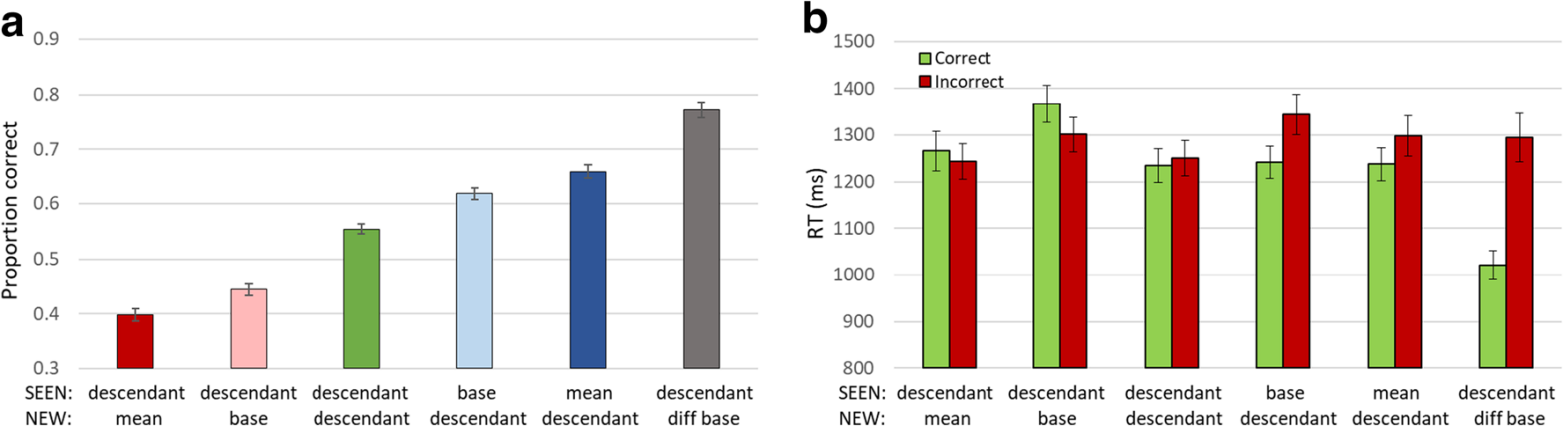
Experiment 2. (**a**) Accuracy of membership task performance for the different trial subtypes, illustrating implicit statistical effects of mean (dark blue and red bars vs. baseline green bar), base (light blue and pink vs. green) and range effect (grey vs. green). (**b**) Correct (green) and Incorrect (red) response RTs for each subtype, illustrating absence of speed-accuracy trade-off

The ancestor (base or mean) and range effects seen for low-level parameters, for categories, and for random amoeba shapes (Experiment 1) are confirmed here, as shown in Fig. 7a. Judging by either base or mean, perception and memory of the ancestor is superior to that of descendant shapes (RM-ANOVA: base: F(2,288) = 65.128, p < 0.001; mean: F(2, 288) = 133.192 , p < 0.001), so that when judging which of two test images was present in the trial sequence, participants are better at recognizing the base (0.61 ± 0.011, Fig. 7a, light blue bar) or mean (0.65 ± 0.01, dark blue) rather than another image (0.55 ± 0.009, baseline, Fig. 7a, green bar); and chose this image even when not included in the sequence, whether the base (0.44 ± 0.01, pink bar) or mean (0.39 ± 0.011, red; percent less than 50% means greater choice of the NEW image not included in the sequence). In addition, when the NEW image was from another ancestor, it was easily rejected (F(1,144) = 191.871, p < 0.001), so that performance was high (0.77 ± 0.013, Fig. 7a, grey bar). All result t-tests showed p < 0.001, here and in all comparisons, unless explicitly stated otherwise.

Note that the ancestor effect was greater (t-test, p < 0.001) for the mean (0.65–0.39 = 0.25 ± 0.19; SEEN = mean vs. NEW = mean) than for the base (0.61–0.44 = 0.170 ± 0.16; SEEN = base vs. NEW = base), as might be expected, as the alleged “ancestor” derived by the participants from the sequence elements would be closer to the mean than to the base. Nevertheless, the base effect is also significant, and close to the mean effect, as might be expected since mean and base images are similar.

RTs are shown in Fig. 7b. The general rule of correct responses being faster than incorrect ones is maintained slightly for baseline, and strongly when the NEW image is from a different base, but is overshadowed by the choice of mean or base being faster than choice of the alternative test image. Thus, when the mean or base was in the sequence, correct trial responses are faster than incorrect responses, and when the mean or base was not in the sequence, but appeared as a test image, it was chosen more often that the alternative, and such “incorrect” choices were faster. While this effect is present for both base and mean tests, interestingly, the shift in relative RT for SEEN = base/mean compared to NEW = base/mean was greater for base than mean (t-test: base RT effect p < 0.001; mean RT effect n.s.).

### Experiment 3. Sets of star-like amoeba shapes – one change at a time

Thus far, we have found that viewing sequences of random amoeba (Experiment 1) or star amoeba (Experiment 2) shapes, participants show the same pattern of perceptual results on the subsequent memory test as they do with sequences of low-level parameter or category membership sequences. We now wished to find which star amoeba ancestor-descendant changes have the most influence on perception, and result in the largest mean and base effects. If we change one parameter at a time, which will show the greatest mean and base effects, and which changes will not be perceived as strongly by participants? This was the goal of Experiment 3.

**Fig. 8:**
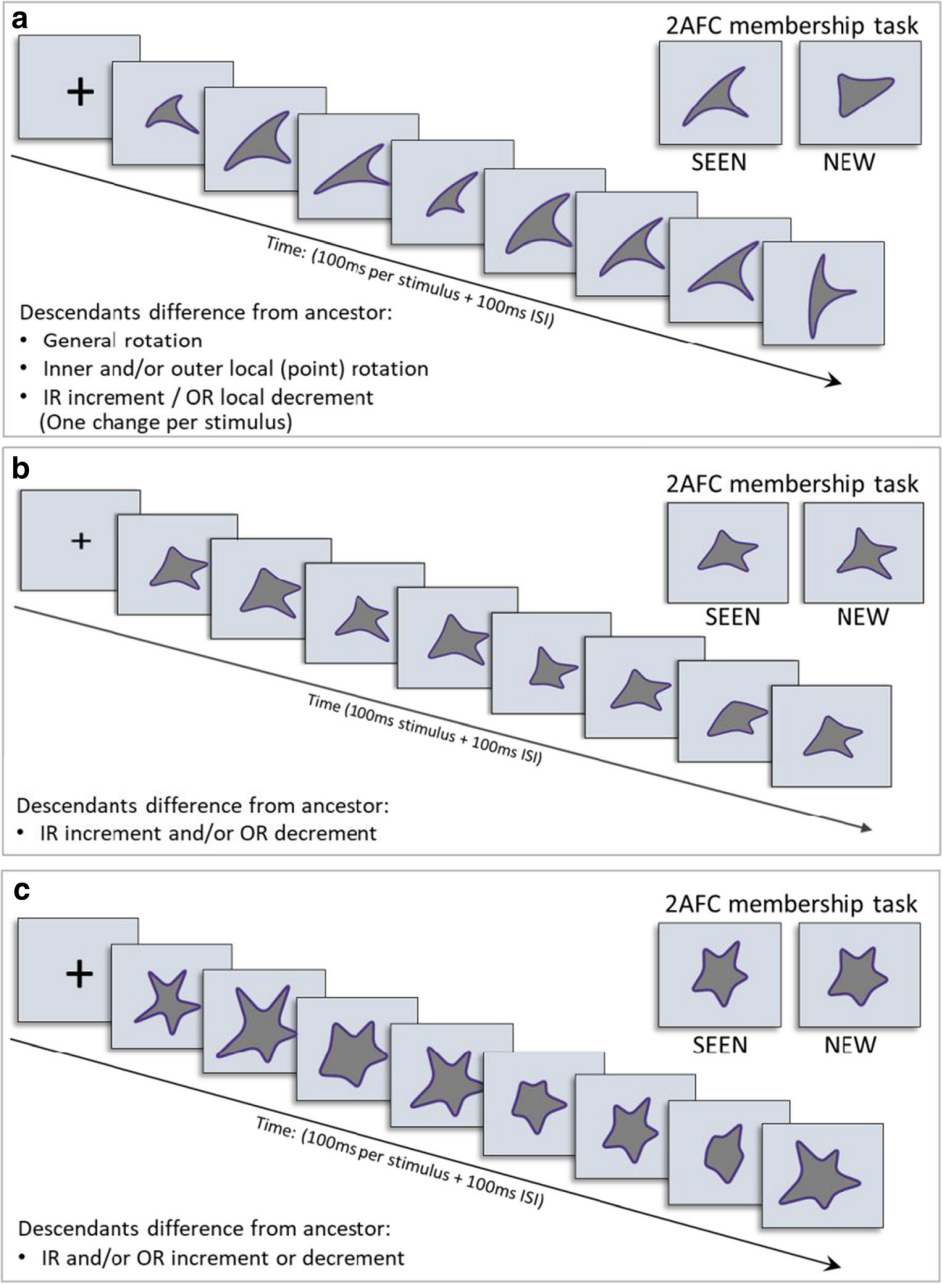
Trial examples for Experiments 3–5. Paradigm like Experiments 1–2, with RSVP sequence (100 ms/shape + 100-ms ISI) followed by a two-alternative forced-choice (2-AFC) membership test. (**a**) Experiment 3. One change/stimulus from the possible three: general rotation, local point rotation, local radius change (IR increase or OR decrease). (**b**) Experiment 4. Descendants created only by local radius changes (IR increase or OR decrease). (**c**) Experiment 5. Symmetric changes for each two descendants included in the RSVP sequence, so that the sequence mean equals the ancestor base. Only local radius changes (IR increase/decrease; OR increase/decrease). In Experiment 5, trials where the ancestor was a sequence member, RSVP had nine stimuli; where not a member, RSVP had eight stimuli

Experimental designs of Experiments 3–5 are like those of Experiments 1–2, with specific changes of descendant variability (differences from ancestor). Modification details are provided in each experiment. Trial designs of Experiments 3–5 are illustrated in Fig. 8.

Experimental paradigm for Experiment 3 was the same as for Experiment 2 (see Fig. 8A), except that now there was only one change in each descendant image from its ancestor, either global image rotation, or star point rotation, or outer or inner point radius change (changing **p**/2 points for even **p**; (**p**+1)/2 for odd **p)**.

#### Results

**Fig. 9:**
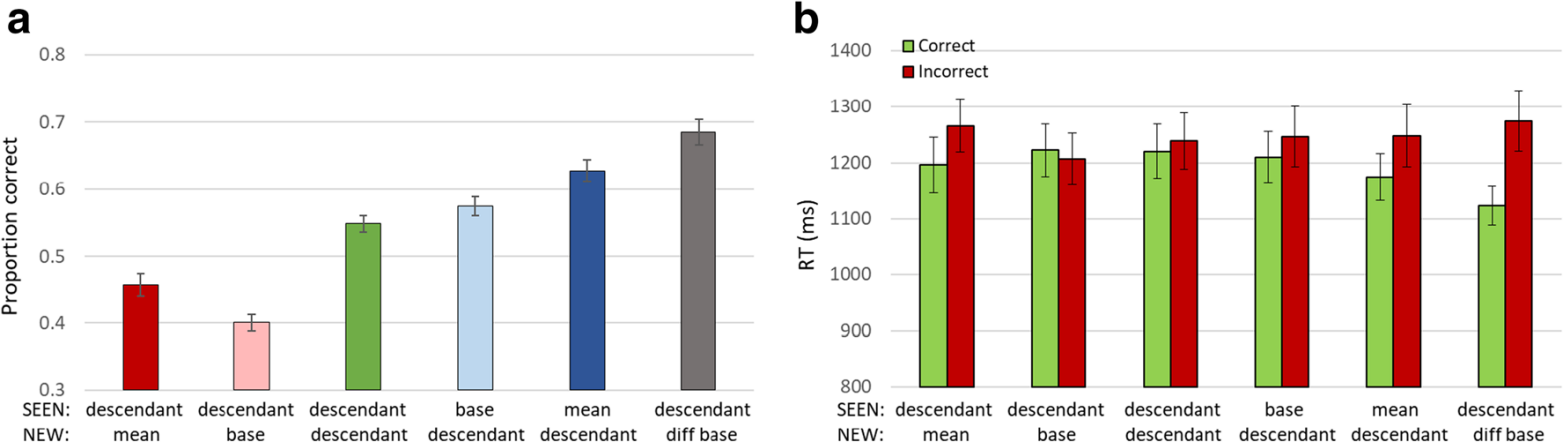
Experiment 3. (**a**) Accuracy of membership task performance for the different trial subtypes, illustrating implicit statistical effects of mean (dark blue-red bars), base (light blue-pink bars) and range effect (green-grey bars). (**b**) Correct and Incorrect reaction times (RTs) for each subtype illustrating no speed-accuracy trade-off (green=correct choices, red=incorrect choices)

The basic ancestor (base or mean) and range effects seen for low-level parameters, and in Experiments 1 and 2 are confirmed here, as shown in Fig. 9a. Judging by either base or mean, perception and memory of the ancestor is superior to that of descendant shapes (base: F(2,178) = 44.742, p < 0.001; mean: F(2,178) = 30.03, p < 0.001), so that when judging which of two test images was present in the trial sequence, participants are better at recognizing the base (0.57 ± 0.014; t-test: p = 0.07, Fig. 9a, light blue bar) or mean (0.62 ± 0.016; t-test: p < 0.001, dark blue bar) rather than another image (0.54 ± 0.012, baseline, green bar); and chose this image even when not included in the sequence, whether the base (0.40 ± 0.012; t-test: p < 0.001, pink) or mean (0.45 ± 0.016; t-test: p < 0.001, red). When the NEW image was from another ancestor, it was easily rejected (F(1,89) = 54.458, p < 0.001), so that performance was elevated (0.68 ± 0.018; t-test: p < 0.001, grey bar). RTs are shown in Fig. 9b. Incorrect choices (red bars) are generally slower than correct choices (green) except for choosing NEW = base, but this preference is not significant (p = 0.32).

Unlike in Experiment 2, here the relative ancestor effect was about the same for mean and base, (0.57–0.40 = 0.62–0.45 = 0.17 ± 0.02 in both cases; t-test SEEN vs. NEW = base or mean p < 0.001; t-test SEEN = base minus NEW = base vs. SEEN = mean minus NEW = mean, p = 0.47), reflecting an equal asymmetry in that both NEW = base < NEW = mean and SEEN = base < SEEN = mean, though each inequality is significant (i.e. performance in trials where the NEW shape is the base shows lower accuracy vs. where it is the mean; and performance when the SEEN shape is the base shows lower accuracy vs. when it is the mean; p < 0.01 in both cases).

**Fig. 10:**
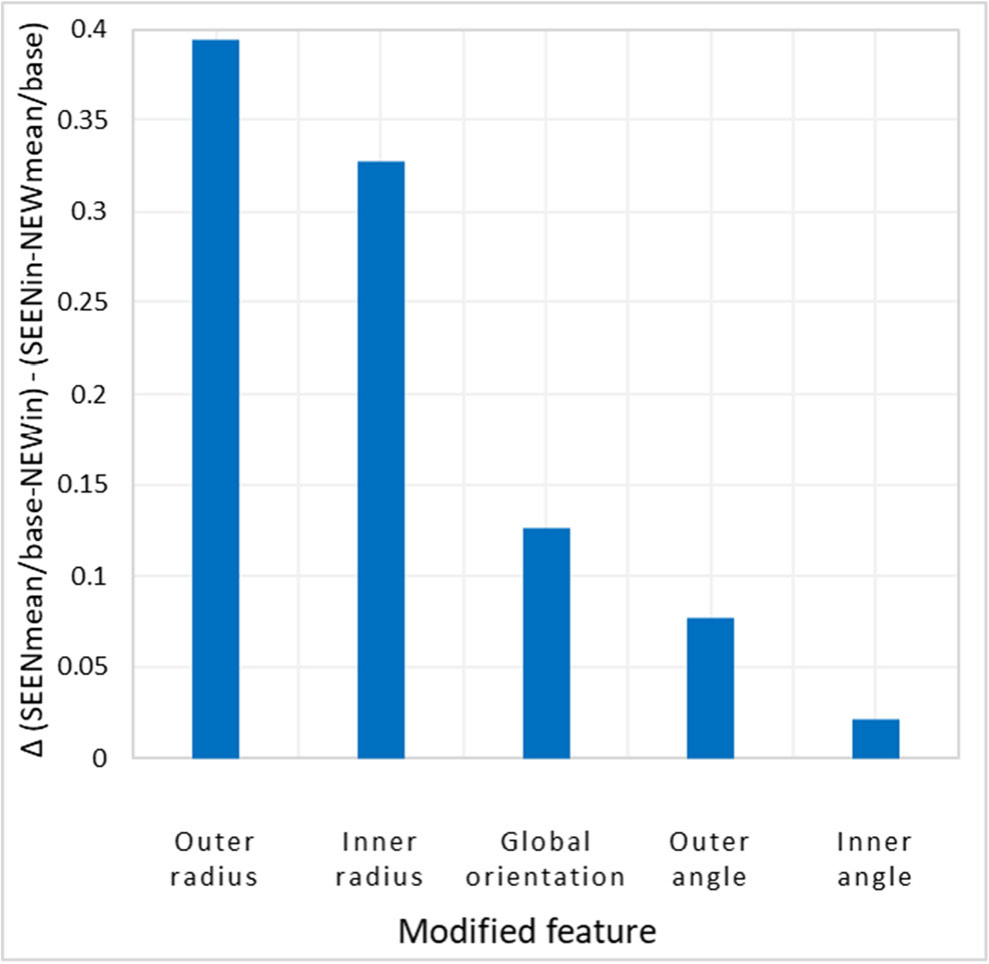
Relative contribution of each feature modification to mean and base effects. The contributions were evaluated by a measurement of both mean and base effects (subtraction of trials wherein mean/base were SEEN shapes from trials where mean/base were NEW shapes in the two-alternative forced-choice (2-AFC) membership tasks). These effects were calculated separately for each feature modification of the NEW and SEEN shapes and plotted here

The main goal of this experiment was to compare the effects of different changes in the ancestor to create the descendants. In Fig. 10 we plot the average of the mean and base effects for each ancestor-to-descendant modification, i.e. accuracy when SEEN = mean/base minus accuracy when NEW = mean/base. The two largest effects were changes of inner or outer radius, which were significantly larger than the effects of the other modifications, namely, global rotation, and outer or inner angle shifts. We therefore decided to concentrate on these larger effects in the following experiment.

### Experiment 4. Sets of star-like amoeba shapes – radius changes

The goal of Experiment 4 was to vary the degree of radius change to quantify the amoeba shape mean and base effects and their dependence on the degree of change. We wish to plot accuracy as a function of the relative distance of the test images from the ancestor, as reflected in the mean or base shape, like the plots in Fig. 3c and d for low-level features and category typicality.

The procedures were identical to those of Experiment 3, creating descendants by introducing only one change from the ancestor. The only difference was that here the change was only in the outer or inner radius of some of the star points, (again changing p/2 points for even p and (p+1)/2 for odd p). Each varied point of the star-like shape was increased in inner radius and/or decreased in outer radius. The change could be small or large for each; outer radius could be 1, 0.8, 0.6 of base, and inner radius could be 1, 1.25, 1.5 of base (excluding, of course, the case where both are 1.0, which is the base itself).

#### Results

**Fig. 11:**
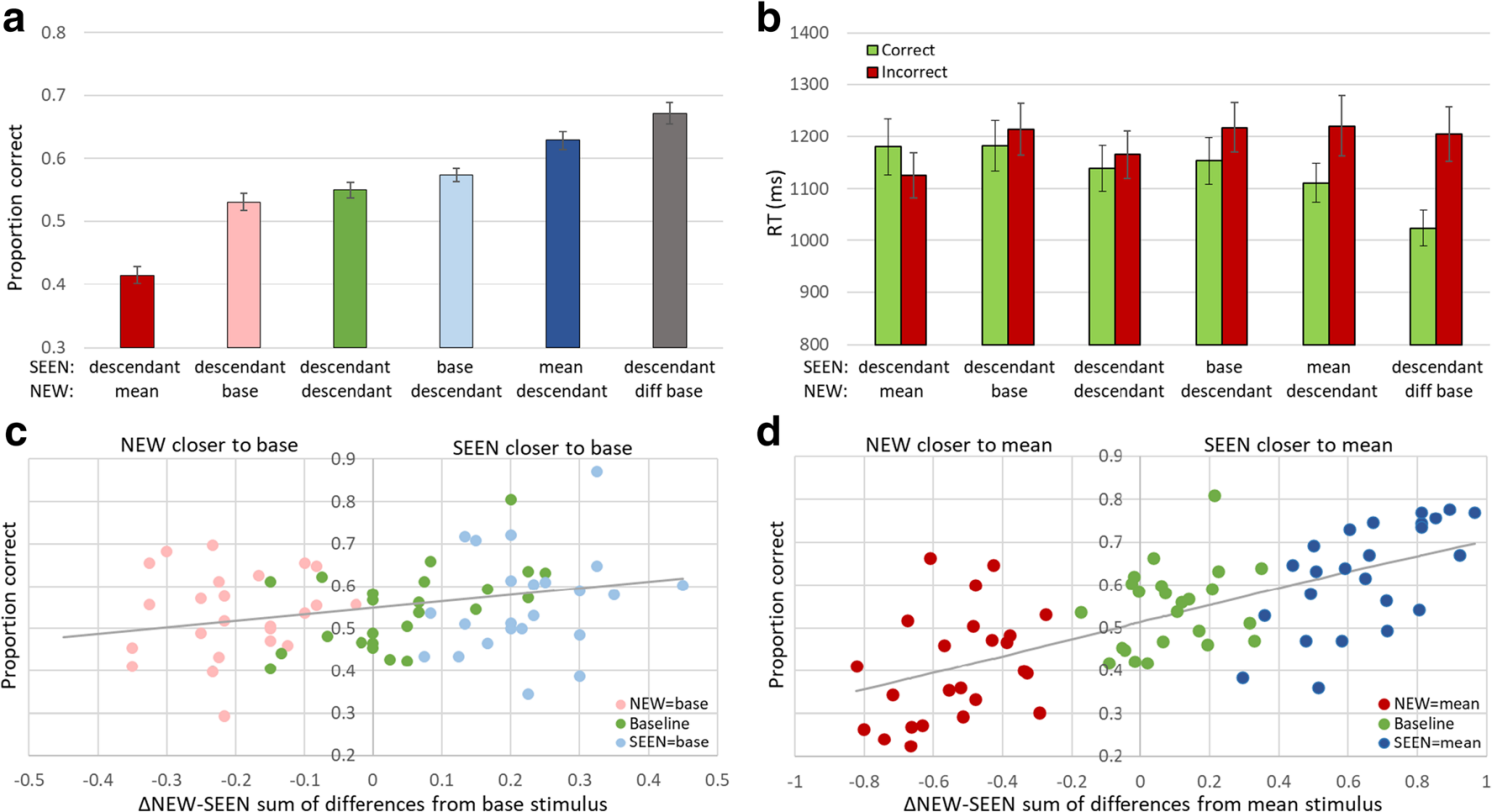
Experiment 4. (**a**) Accuracy of membership task performance for different trial subtypes, illustrating implicit statistical effects of mean (dark blue-red bars), base (light blue-pink), and range effect (grey) compared to baseline (dark green). (**b**) Reaction times (RTs) for trials with correct (light green) and incorrect (red) responses for each subtype illustrating lack of speed-accuracy trade-off. (**c**) Graded base effect. (**d**) Graded mean effect. X-axis in C and D is calculated difference between distances of the two test shapes from base or mean, respectively. Negative values represent trials where NEW is closer to mean or base, positive values where SEEN is more like mean or base. Dot colors represent trial subtypes as in (**a**)

The basic ancestor and range effects seen for Experiments 1 , 2 and 3 were found here, too, as shown in Fig. 11. Judging by either base or mean, perception and memory of the ancestor is superior to that of descendant shapes (RM-ANOVA: base: F(2,180) = 2.98, p < 0.001; mean (F(2,180) = 54.08, p < 0.001). Participants were only slightly and insignificantly better at recognizing the base (0.57 ± 0.01; t-test: p = 0.15, Fig. 11a, light blue bar), but highly significantly in recognizing the mean (0.62 ± 0.014; t-test: p < 0.001, dark blue bar) rather than another image (baseline: 0.55 ± 0.012, green) in membership judgments. Similarly, they chose this base even when not included in the sequence slightly below the baseline (0.53 ± 0.014; t-test: p = 0.068, pink bar), and the mean significantly below the baseline (0.41 ± 0.013; t-test: p < 0.001, red). In addition, when the NEW shape was from another ancestor, it was easily rejected (F(1,90) = 35.43; p < 0.001), so that performance was: 0.67 ± 0.016; p < 0.001, grey bar. In addition, RTs are shown in Fig. 11b. Incorrect choices are generally slower than correct choices with the one exception of choosing NEW = mean (t-test RT mean effect p < 0.01). Unlike in Experiment 3, here the relative ancestor effect was more robust for the mean (mean: 0.21 ± 0.02, t-test p < 0.001; base: 0.04 ± 0.01, t-test p < 0.05), reflecting the more robust effect of choosing the mean (t-test SEEN = base minus NEW = base vs. SEEN = mean minus NEW = mean, p < 0.001).

To evaluate the graded base and mean effects, the relative distance of test descendants from the ancestor base or ensemble mean, we calculated the sum of distances from the base or mean in all inner and outer radii for both the NEW and the SEEN test shape. We then subtracted the difference of the SEEN to the base or mean from the difference of the NEW from the base or mean. Thus, negative values indicate that the NEW was more similar to the base or mean relative to the SEEN, and vice versa for positive values (x-axes of Fig. 11c and d). This measure, adopted from previous studies (Khayat & Hochstein, 2018, 2019a; see Fig. 3), enabled us to investigate base or mean perception at a higher resolution, testing not only the response when either SEEN or NEW stimulus is precisely the base or mean, but how will participants respond in the membership task when neither test image is the base or mean, but one of the two alternatives is closer to the mean than the other. We find that performance was elevated gradually as a function of resemblance of the SEEN shape to the base (Fig. 11c) or mean (Fig. 11d) relative to the NEW shape resemblance to the base or mean (Pearson correlation – base: r = 0.293, p < 0.05; mean: r = 0.697, p < 0.001).

Limiting the ancestor-to-descendant change to changes in outer and inner radius, and having two levels of change for each, allows us to measure the influence of relative distance from the base or mean of the SEEN versus NEW test element. When the SEEN test element is the base or mean, the relative distance is just the distance of the NEW element from the base or mean (light and dark blue points in Fig. 11c and d, respectively), and when the NEW test element is the base or mean, the relative distance is just the distance of the SEEN element from the base or mean (pink and red points in Fig. 11c and d, respectively). When neither is the base or mean, and both derive from the same ancestor (green points in Fig. 11c and d), we have the mid-range points, which together form the baseline result (SEEN and NEW both descendants).

### Experiment 5. Sets of star-like amoeba shapes – mean = base = ancestor

Procedures were the same as in Experiment 4 except that here we made sure that the mean value of the RSVP sequence elements was equal to the ancestor star-amoeba shape. As in Experiment 4, each shape stimulus varied from the ancestor base in increased or decreased inner and/or outer radius, but here the changes could be in both directions, and the difference in one ensemble stimulus was balanced by using the opposite difference in another stimulus. The changes were thus symmetric, leading to the ensemble mean being equal to the ancestor base. The change in radii (for p/2 points for even p and (p+1)/2 for odd p) were as follows: outer radius 1.4, 1.2, 1, 0.8, 0.6 times base; inner radius 0.5, 0.75, 1, 1.25, 1.5 times base. To maintain the ancestor as the mean of each RSVP trial, when the ancestor was a member of the sequence (SEEN), the RSVP included nine shape stimuli (ancestor + four pairs of descendants). When the ancestor was not a member, RSVP included eight shape stimuli (four pairs of descendants).

#### Results

**Fig. 12:**
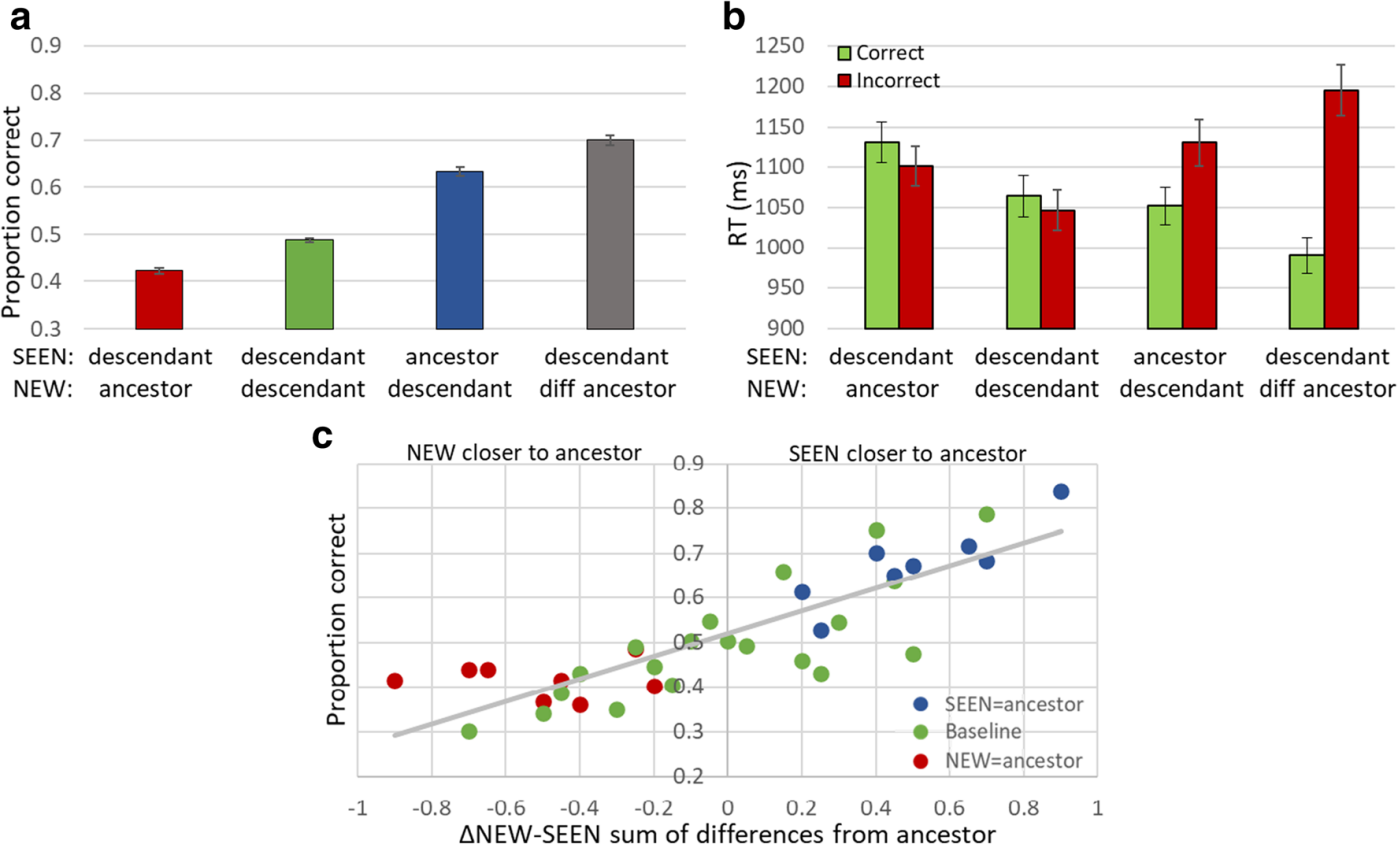
Experiment 5. (**a**) Accuracy of membership task performance for the different trial subtypes, illustrating implicit statistical effects of ancestor (dark blue-red bars) and range effect (grey-green bars). Difference is highly significant (p < 0.001) in both. (**b**) Correct (green) and Incorrect (red) trial reaction times (RTs) for each subtype illustrating no speed-accuracy trade-off, and a flip between speed of correct and incorrect responses between trials of NEW = base and baseline to SEEN = base and NEW = out. (**c**) Graded ancestor effect: X-axis as in Fig. 11c and d; dot colors represent trial subtypes as in (**a**)

The ancestor (mean and base) and range effects seen for previous Experiments 1–4 were measured here, as shown in Fig. 12. Implicit perception and memory of the ancestor shape was highly significant (RM-ANOVA: F(2,398) = 185.667, p < 0.001). Participants tended to select the ancestor whether it was a SEEN (0.63 ± 0.009, Fig. 12a, blue bar) or a NEW (0.42 ± 0.006, red) shape rather than another shape (0.48 ± 0.004, baseline, green) in membership judgments (t-test for both: p < 0.001). This bias was also reflected by the robust ancestor effect (SEEN = ancestor minus NEW = ancestor: 0.63–0.42 = 0.21 ± 0.01). When the NEW shape was from another ancestor, it was easily rejected (F(1,199) = 260.286, p < 0.001), resulting in high performance (0.7 ± 0.011; t-test: p < 0.001, grey bar).

Figure 12b shows reaction time results. As expected, correct (green) responses are generally faster than incorrect responses (red). This trend is reversed for trials where the NEW shape is the ancestor (t-test p < 0.01; t-test RT mean/base effect p < 0.001). It is also seen for baseline trials, but not significantly so. RT results show there is no speed-accuracy trade-off.

Graded ancestor effect, shown in Fig. 12c, was evaluated using the same methods as in Fig. 3c and d (Khayat & Hochstein, 2018, 2019a) and Experiment 4 (Fig. 11c and d). This bias to the shape that is closer to the ancestor confirms our prediction regarding the relative and graded ancestor effect, suggesting that the implicit perception is not to the exact ancestor shape, but it is impacted by relative resemblance. In addition, the finding that when presented with two test images, participants intuitively choose the image with lower distance from the ancestor, is consistent with the suggestion that images are represented as ancestor plus distance from the ancestor. This is consistent with the model of Benna and Fusi (2019), suggesting that we efficiently perceive and store new objects in memory using a compressed representation – by encoding only the difference from the known ancestor object.

## Discussion

### Summary

The threefold goal of these experiments was achieved:

1. We had found that mean perception is automatic, implicit, and on-the-fly for low-level stimulus parameters (size, orientation, brightness; Khayat & Hochstein, 2018). We had also found the same prototype effect phenomenon for categorization (Khayat & Hochstein, 2019a). Since we had only tested well-known categories, acquired over a lifetime (e.g., animals, food, furniture) we wished to bridge these findings by testing for the mean effect for novel categories that could be acquired on-the-fly on a trial-by-trial basis. The amoeba shapes presented an excellent opportunity for this test. Participants indeed learned and perceived the mean or ancestor of the set of amoeba shapes on-the-fly for each trial and used this mean in choosing which of two test images had been seen in the RSVP sequence. This finding strengthens the implication that a similar mechanism underlies mean perception and categorization. While there are other potential causes of this similarity, such as shared source of internal noise, we believe the most parsimonious explanation is common or analogous mechanisms.
2. For low-level mean perception, we could measure the graded effect of relative proximity of the two test images from the mean. For categorization, this was more complex, and we used results of an auxiliary experiment, where we measured category exemplar typicality (using RT when indicating category membership). We wished to use categories where the distance from prototype – or base/mean – was directly measurable. This was made possible by using graded changes from the ancestor star-amoeba shape. We found graded dependences on relative distance from the mean or base shape (Figs. 11 and 12), like those found for the low-level mean effects (Fig. 3c) and that for known categories (Fig. 3d). This finding widens the range of ensemble statistics perception, and establishes a measure for the effect in different scenarios. It confirms the methodology used for measuring category exemplar typicality. It also further strengthens the above suggestion of similar mechanisms for ensemble perception and categorization.
3. Benna and Fusi (2019) suggested that memory capacity can be increased by considering object correlations and that efficient representation is by storing the identity of the appropriate “ancestor” of each pattern, and the difference between it and each “descendant.” We wished to determine if using stimulus patterns related in ancestor-descendant fashion leads to automatic perception of the ancestor pattern. We found that presenting sequences of patterns leads to participants perceiving the mean/base/ancestor of the sequence patterns, supporting the Benna and Fusi (2019) theory. Note, however, that the ancestor derived from the sequence was not necessarily the ancestor which we used to create the descendants. This is because the derivation was ambiguous. The pattern most often preferred by participants was just the mean pattern – which could just as well (or better) have been the ancestor. Significantly, choice of the test image that is closer to the ancestor suggests that these test images, too, are represented by their ancestor and the distance from the ancestor, enabling the choice of the image closer to the ancestor.

Together with the analogy or possible relationship of ensemble statistics perception and categorization, the stimuli of the experiments in the current study may be considered as intermediate level stimulus images, as they are constructed by integrating multiple visual features. Ensemble statistics was shown to be extracted from visual low-level (orientation, brightness) and high-level features (face expression, biological motion) in many studies. The neural correlates underlying these processes remain vague. Though the Para-hippocampal Place Area (PPA) was associated with such processes, as found by Cant and Xu (2012, 2015, 2017), there is no differentiation of low- and high-level ensemble processing. Moreover, the mechanism of this unique processing is yet to be well understood, and a main question to be investigated in future research is – how and where are different ensembles processed? Is there a single domain specializing in that kind of statistical extraction which communicates with specific regions that are selective to the relevant ensemble features (e.g., V1 for line orientation, FFA for faces)? Alternatively, does each perceptual region independently process ensembles that fits its receptive field structure? These questions need to be targeted to understand the strategy and nature of the mechanisms underlying ensemble statistics perception.

## Conclusion

On the fly categorization shows the same category/prototype effects as low-level and categorization experiments, suggesting similar mechanisms might underlie all three, and the jump from low-level parameters to high-level cognitive/semantic categorization is warranted.

Thus, mean perception is found for four perceptual levels: low level (size, orientation, brightness), high level (faces, numbers, lifelikeness), shape categories (star-like, amoebas), familiar categories (fruit, cars, animals). Finding identical properties at all these perceptual levels suggests that the similar mechanisms might be used at all perceptual levels.

Benna and Fusi (2019) suggest individual images might be represented as ancestor plus difference-from-ancestor. Thus, in this view, perceiving the mean/prototype/base is not an additional computation performed by the sensory system for the special purpose of averaging or categorizing groups, but is inherent in the process of building the representation of images in the first place.

## Data Availability

The data and materials for all experiments are available upon request from the authors.

## Appendix

### Violin Plots

We present violin figures for the data of Experiments 1–5 with each participant’s result for each subtype (dots), interquartile range (light bars) and the median result (thick horizontal bar). Compare Figs. 5, 7, 9, 11, and 12, which show the average accuracy over participants.
